# Copy number variations primed lncRNAs deregulation contribute to poor prognosis in colorectal cancer

**DOI:** 10.18632/aging.102168

**Published:** 2019-08-22

**Authors:** Huimin Liu, Xiaoyu Gu, Guihua Wang, Ying Huang, Shaoqing Ju, Jianfei Huang, Xudong Wang

**Affiliations:** 1 Department of Laboratory Medicine, Affiliated Hospital of Nantong University, Nantong, Jiangsu, China; 2 Clinical Biobank, Affiliated Hospital of Nantong University, Nantong, Jiangsu, China

**Keywords:** colorectal cancer, copy number variations, Long non-coding RNAs, prognosis

## Abstract

Copy number variations (CNVs) are crucial genetic change elements in malignancies, and lncRNAs deregulation induced by genomic and epigenomic aberrations plays key driving role in tumorigenesis, including colorectal cancer (CRC). However, effects of CNVs associated with lncRNAs in CRC is largely unknown. Here, we perform integrative analysis considering messenger RNA expression levels, DNA methylation and DNA copy numbers from 289 cases of CRC specimens. There are five prognostic subtypes of CRC determined by multi-omics integration, and differentially expressed lncRNAs (DE-lncRNAs) are acquired among five subtypes and normal cases. Finally, CNVs pattern matched with DE-lncRNAs reveals a signature including 10 lncRNAs (LOC101927604, LOC105377267, CASC15, LINC-PINT, CLDN10-AS1, C14orf132, LMF1, LINC00675, CCDC144NL-AS1, LOC284454), conspicuously contributing to poor prognosis in CRC, which can be validated in another independent dataset. Together, our research is interested in copy number changes relevant with lncRNAs, not only expending the spectrum of CNVs, but also perfecting the regulation network of lncRNAs in CRC. The main purpose is to provide novel biomarkers for prognostic managements of CRC patients.

## INTRODUCTION

Colorectal cancer (CRC) is the second leading cause of cancer-related death among all malignancies worldwide [1]. Despite surgical operation, radiotherapy, chemotherapy and personalized medicine have strongly prolonged survival of CRC patients, the 5-year relative survival remains less than 50% [2]. In view of genomic variations, epigenomic alterations and transcriptional deregulation, CRC is often signatured with heterogeneous behaviors and a couple of molecular subtypes have been proposed, which describe detailed pictures of tumor biology mechanisms.

Copy number variation (CNV) is defined as a pattern of genetic structural variation, and generally refers to the increase or decrease in copy number of genomic fragments between 1 kb and 3 Mb [3]. Copy number amplifications or deletions in cancer genomes often induce oncogenes expression or deactivation of tumor suppressor genes and harbor significant influence on cellular functions, including adhesion, recognition, communication [4, 5]. Increase in copy number of IGHG3 located at 14 chromosome often contributes to overexpression of this gene and high prevalence and mortality in prostate cancer [6]. Not only referring to tumor biology, CNVs are also familiar with dosage effects in some intricately immunological diseases. Enhanced HIV/acquired immunodeficiency syndrome (AIDS) susceptibility is visible in individual who has CCL3L1 copy number aberrations [7]; CNV of human Fcgr3 gene is a determinant of glomerulonephritis [8]; Low copy number of component C4 is a danger factor and high copy number is a shielding factor in systemic lupus erythematosus (SLE) [9]. In the light of these expounded findings, bulks of investigations have shed light on phenotypic changes and tumor progression caused by CNVs, concentrating on messenger RNA (mRNA), but few studies explicate the regulatory relationship between CNVs and non-coding RNAs, especially lncRNAs. With this concern in mind, here, we are particularly interested in taking a research on the relationship between structural variations in whole genomes and lncRNAs in CRC.

Long non-coding RNAs (lncRNAs) are known as size larger than 200nt without protein coding ability [10]. Since their discovery, increasing numbers of lncRNAs serve as significant modulators in tumorigenesis and progression in human CRC [11–14]. Furthermore, lncRNAs have also come forward as potential prognostic biomarkers in CRC, and survival analysis indicates that aberrant lncRNAs expression hold increasing risk of relapse [15–17]. However, there are few attentions calling for potential prognostic biomarkers by means of detecting DNA copy number amplifications or deletions of lncRNAs.

In this research, we could identify five molecular subtypes associated with prognostic outcomes of CRC patients based on messenger RNA expression levels, DNA methylation and DNA copy numbers. R package-DEseq2 was employed to distinguish differentially expressed mRNAs and lncRNAs across five molecular subtypes and normal tissues. Despite the well-known effect of CNVs on transcriptomic regulation, it is still unclear whether CNVs is systematically related to the expression levels of lncRNAs in CRC. By analyzing copy number profiles of the whole genome lncRNAs, we investigated these deregulated lncRNAs induced by copy number amplifications or deletions. Furthermore, prognostic-related lncRNAs were exploited for Kaplan-Meier (KM) survival analysis. Overall, we are aimed at identifying CNVs-related lncRNAs guiding prognosis in CRC.

## RESULTS

### Multi-omics integration analysis

**Figure 1:**
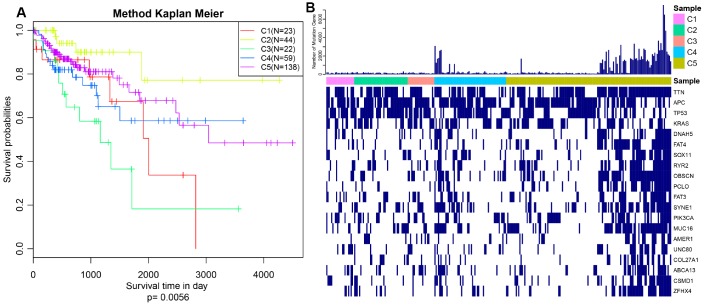
**Prognosis description and profiling of mutation genes across five subtypes.** (**A**) Kaplan–Meier plot analysis for five subtypes identified by iCluster (C1, C2, C3, C4, C5) is shown for overall survival (OS). (P=0.0056) (**B**) Exhibition of top 20 mutated genes among five molecular subtypes.

The expression profiles of PCGs, CNVs and 450k methylation were combined with prognosis status based on univariate cox proportional hazards model. Eventually, 2118 genes, 5015 CNV regions and 7083 CpG sites were obtained with the significant threshold p<0.05. Subsequently, 289 CRC patients sharing in three omics were classified into 5 molecular subtypes using iCluster. Five kinds of subtypes C1, C2, C3, C4, C5 consisted of 23, 44, 23, 60, 139 CRC patients respectively (Table 1). C3 group was harboring the worst survival probabilities (overall survival), and C2 subtype owned the optimal prognosis status (p=0.0056) (Figure 1A). Progression free survival (PFS) among five subtypes was almost consistent with overall survival, similarly, C3 group got hold of the worst survival probabilities (PFS) (Supplementary Figure 1). The distribution of TNM stage among five subtypes clearly showed that the proportion of advanced patients (stageIII+stageIV) in C3 group was much larger than other four subtypes accounting for about 60% (Supplementary Figure 2). Simultaneously, we described the spectrum of gene mutation status among five subtypes, and selected the top 10 genes with the highest mutation rate in each subtype. There are total 20 mutation related genes acquired and this phenomenon highlighted that the most common mutation related genes in five subtypes had higher coincidence. We made further observations revealing that these 20 genes were differently distributed in five subtypes and the mutation frequency of the same gene in different subtypes is also distinguished (Figure 1B). Obviously, the frequency of gene mutations in C5 subtype was significantly higher than other subtypes, and even in some C5 subtypes samples, these 20 gene mutations almost existed. In addition, TTN, APC, TP53 and KRAS mutations were especially more common than other genes, which was highly shining upon that these four genes mutations may imply pivotal role in carcinogenesis. These findings suggested that our strategy for subtypes classification based on the expression profiles of PCGs, 450k methylation and CNVs data could predict distinct prognostic situation of CRC and feature certain regulatory relationship among genomic, epigenomic and transcription level.

**Table 1:** Identification of five subtypes and the distribution of CRC samples across subtypes.

**Cluster**	**SampleCount**
C1	23
C2	44
C3	23
C4	60
C5	139

### Differentially expressed lncRNAs and mRNAs across subtypes

**Figure 2:**
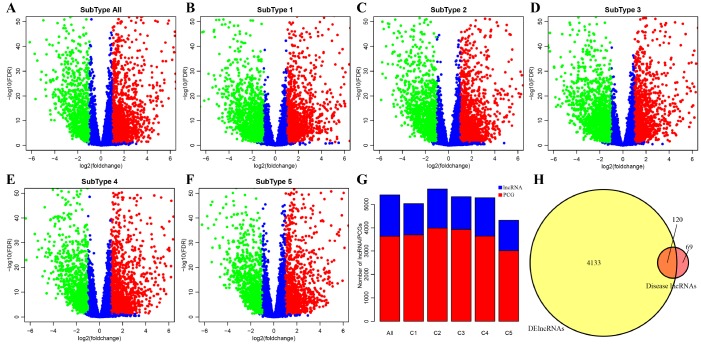
**Identification of key differentially expressed protein-coding genes and lncRNAs among five subtypes.** (**A**) Volcano plot shows the differential expression pattern of lncRNAs between tumors and adjacent normal tissues (named “subtype all”). The upregulated genes are shown as red and the downregulated genes are shown as green. (**B**–**F**) The up-(red) and down-(green) regulated lncRNAs across five molecular subtypes (subtype 1, 2, 3, 4, 5) are also shown as volcano plots, respectively. (**G**) Distribution of DE-lncRNAs and DE-PCGs among five subtypes (C1, C2, C3, C4, C5) and tumors and adjacent normal tissues (named “subtype all”) are shown. LncRNAs are presented as blue and PCGs are shown as red. (**H**) Venn diagram displays the intersection of DE-lncRNAs and Disease lncRNAs. Total 120 overlapped lncRNAs are indicated. Remaining 69 disease lncRNAs are excluded in our research.

**Figure 3:**
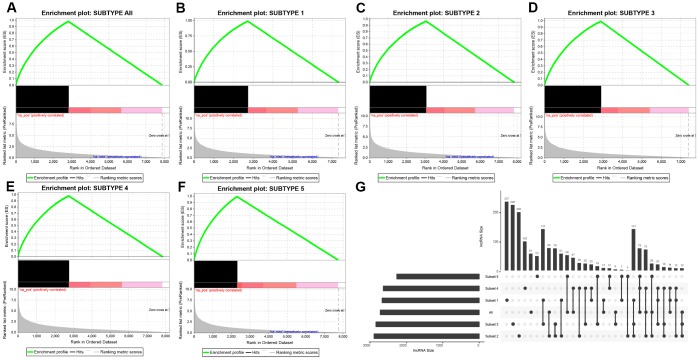
**Distribution condition of DE-lncRNAs in subtypes.** (**A**–**F**) GSEA analysis shows different enrichment states among these five subtypes (subtype 1, 2, 3, 4, 5) and tumors and adjacent normal tissues (named “subtype all”) based on difference multiple. The enriched lncRNAs are mainly focused on the left (presented as black bulks), which referring to larger difference multiple. (**G**) The overlapped lncRNAs exist in subtypes. The dot represents subtype and the line represents the overlapped lncRNAs across subtypes. LncRNA size points to the amount of DE-lncRNAs.

DE-lncRNAs in five subtypes and carcinoma and normal tissues were 2668, 2890, 2838, 2634, 2265, 2718, respectively (Table 2). DE-PCGs in five subtypes and carcinoma and normal tissues were 3702, 3989, 3934, 3647, 3013, 3642, respectively (Table 2 and Figure 2G). It was evidently exhibiting that C5 subtype was holding less differential mRNAs and lncRNAs, on the contrary, C2 and C3 subtypes were holding more differential mRNAs and lncRNAs than other subtypes. A total of 4253 DE-lncRNAs and 5808 differential expression of PCGs were obtained. The upregulated lncRNAs were remarkably more than down-regulated lncRNAs (Figure 2A–2F). Whereafter, we downloaded lncRNAs closely related to disease from the LncRNA Disease and Lnc2Cancer database and 611 lncRNAs were obtained, which were compared to 4253 subtypes specific lncRNAs (Figure 2H). There were 120 lncRNAs contained in previous DE-lncRNAs and the significance of these lncRNAs was tested by hypergeometric test (p=0.003929) (Supplementary Table 1). In order to understand the clustering situation of these DE-lncRNAs, gene set enrichment analysis (GSEA) was employed based on fold change of each lncRNA in five molecular subtypes and carcinoma and normal tissues (Figure 3A–3F). The results indicated that these DE-lncRNAs were always clustered in the gene set with larger difference multiples. There was almost no intersection of lncRNAs between all five molecular subtypes and carcinoma and normal tissues. Most lncRNAs were merely enriched in one subtype, such as, subtype 1 having 237 lncRNAs; subtype 2 having 202 lncRNAs; subtype 3 having 226 lncRNAs; subtype 4 having 102 lncRNAs; subtype 5 having 53 lncRNAs and carcinoma and normal tissues having 61 lncRNAs, respectively. The remaining lncRNAs were focused on two kinds of subtypes or three kinds of subtypes. It was obviously seen carcinoma and normal tissues shared a great deal of lncRNAs with other subtypes (Figure 3G). This phenomenon highly suggested that these identified lncRNAs are distinctively enriched in specific molecular subtypes.

**Table 2:** Differentially expressed protein-coding genes (DE-PCGs) and lncRNAs (DE-lncRNAs) between tumors and adjacent tissues (subtype All) and five subtypes (C1, C2, C3, C4, C5).

**Type**	**C1**	**C2**	**C3**	**C4**	**C5**	**All**
PCG_Down	1843	1696	1996	1401	1296	1308
PCG_Up	1859	2293	1938	2246	1717	2334
PCG_All	3702	3989	3934	3647	3013	3642
Lnc_Down	1329	1217	1439	993	952	954
Lnc_Up	1339	1673	1399	1641	1313	1764
Lnc_All	2668	2890	2838	2634	2265	2718

### Co-expressing modules between lncRNAs and PCGs based on WGCNA

**Figure 4:**
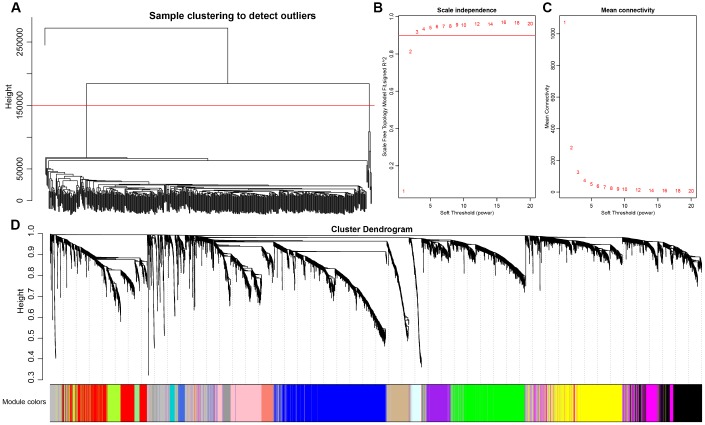
**Weighted gene co-expression network analysis (WGCNA) to identify clinical modules.** (**A**) Hierarchical clustering is applied to exclude some outliers samples. Objects with height greater than 150000 (upper the red line) are excluded. (**B**–**C**) Analysis of network topology for soft thresholding powers. The red line represents the square of correlation coefficient reaches to 0.9. The mean connectivity under different values of ‘power’ are shown. (**D**) Modules dendrogram of samples and modules are indicated by different colors.

Co-expression between lncRNAs and PCGs was analyzed by hierarchical clustering. We defined the samples with distance more than 150000 as outlier samples for screening, and finally got 492 CRC cases for co-expressing analysis (Figure 4A). We accepted 3 as the final soft threshold in order to ensure the network belonging to non-scale property (Figure 4B, 4C), which can effectively strengthen strong correlation and weaken weak correlation or negative correlation. Next, the expression matrix was transformed into adjacency matrix, and then the adjacency matrix was transformed into topological overlap matrix (TOM) for reduced noise and false correlation. According to the dynamic tree cut criterion linked with TOM structure, the threshold for module partitions was constructed at 30. After determining the initial modules, the next was to establish new modules presenting as different colors, including calculating specific eigengenes of each module; clustering analysis of each module; grouping close modules into one new module with height = 0.25, deepsplit = 2, minModuleSize = 30. A total of 27 modules (Figure 4D) were eventually obtained and the module of grey was signatured with genes which were unable to be classified into other modules. P value represents the significant aggregation degree and fold change represents the aggregation multiple. There was no significant difference between lncRNAs and PCGs among 27 modules (Table 3).

**Table 3:** Coordination expression of PCGs and lncRNAs in 27 modules.

**Module**	**All**	**Lnc**	**PCG**	**p.value**	**fc**
green	697	299	398	0.378808	1.025934
cyan	139	58	81	0.584449	0.977854
tan	205	84	121	0.672971	0.948037
magenta	350	149	201	0.474915	1.012328
blue	1083	436	647	0.92705	0.920266
greenyellow	222	104	118	0.09268	1.203601
brown	908	378	530	0.671729	0.973974
purple	248	103	145	0.618262	0.970064
yellow	726	303	423	0.633562	0.978213
pink	390	163	227	0.596578	0.980602
royalblue	70	30	40	0.506279	1.024218
darkgrey	48	20	28	0.588628	0.975446
turquoise	1253	523	730	0.669036	0.978386
black	464	201	263	0.336854	1.04369
lightcyan	99	44	55	0.366243	1.092499
darkturquoise	51	19	32	0.807053	0.810839
grey60	77	34	43	0.410796	1.079796
salmon	142	64	78	0.275304	1.120512
white	36	16	20	0.458678	1.092499
darkorange	41	22	19	0.093987	1.581249
orange	41	16	25	0.716994	0.874
lightyellow	72	32	40	0.397353	1.092499
darkgreen	63	31	32	0.16111	1.322949
red	521	211	310	0.812304	0.929506
darkred	66	24	42	0.864677	0.780357
lightgreen	73	32	41	0.437124	1.065853
midnightblue	109	44	65	0.690877	0.924423

**Figure 5:**
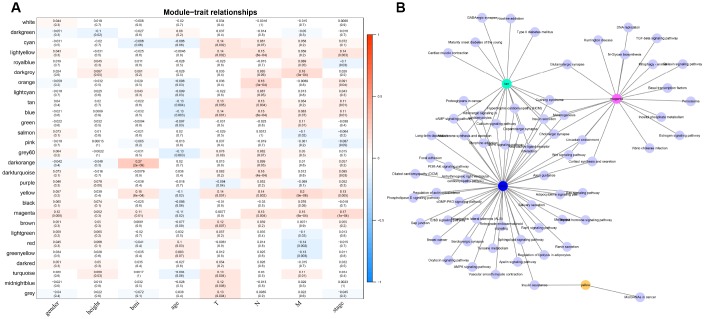
**The correlation analysis between modules and clinical factors.** (**A**) Correlation heat map shows the association between 27 modules and CRC clinical factors (gender, height, BMI, age, T, N, M, stage). The vertical axis represents these identified 27 modules and the horizontal axis corresponds to involved clinical factors, respectively. The corresponding correlation and p value are shown in the first line and the second line respectively. (**B**) The network topology shows enrichment pathways among four phenotypical related modules.

**Figure 6:**
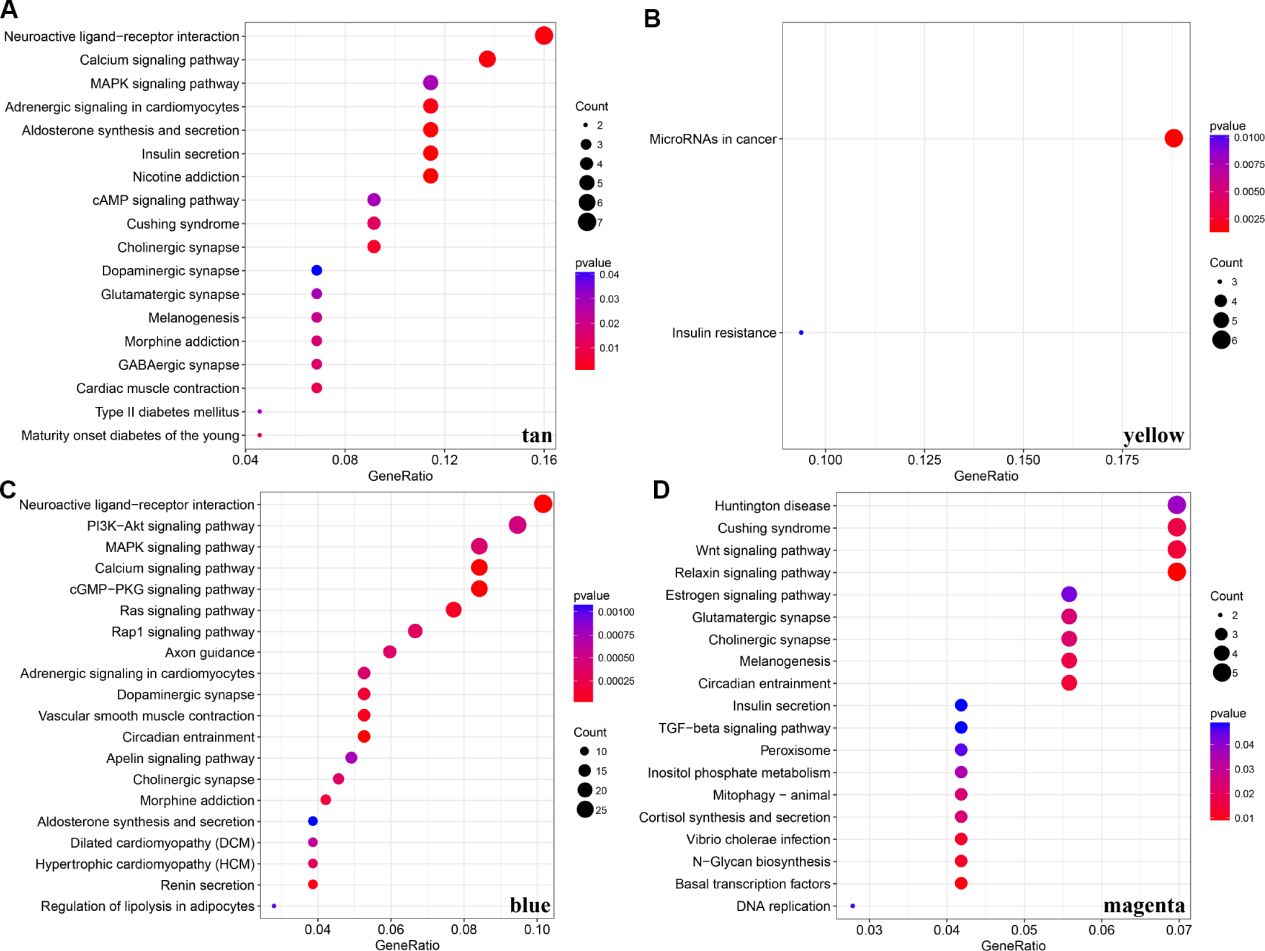
**KEGG pathway enrichment analyses for genes existing in these four modules (tan, yellow, blue, magenta).** (**A**–**D**) KEGG pathway enrichment analyses for significant pathways in modules, including tan, yellow, blue, magenta. KEGG: kyoto encyclopedia of genes and genomes

For the interest of finding biologically associated modules, we need to correlate modules with external information, for instance clinical data of array information and functional enrichment analysis of gene information. Therefore, we executed correlation analysis by combining patients’ gender, age, height, weight, BMI, T, N, M, stage with each module (Figure 5A). What could be clearly indicated in the correlation analysis that all modules except white, pink, black, darked were related to at least one phenotype. Besides, four modules: tan, blue, yellow, magenta were finally selected with more than three kinds of phenotypic correlations for functional enrichment analysis and the significant enrichment pathways p<0.05 were picked up. These four kinds of modules were clustered in distinguished pathways and the cross-talk between these four modules was missing (Figure 5B), suggesting each module may drive in different biological function. Tan module was associated with 18 pathways (Figure 6A, Supplementary Table 2) and neuroactive ligand-receptor interaction and calcium signaling pathway were in the highest flight in this module. MAPK signaling pathway also occupied superior gene ratio in this module and had been proved holding significant position in CRC-promoting process. Only two pathways were enriched in yellow module, of which microRNAs in cancer may possess outstanding role (Figure 6B, Supplementary Table 3). Besides, blue module enriched in 47 pathways (Figure 6C, Supplementary Table 4), and the remarkable pathways included neuroactive ligand-receptor interaction, PI3K-AKT signaling pathway and MAPK signaling pathway. Genes in magenta module mainly enriched in Wnt signaling pathway, TGF-beta signaling pathway and DNA replication which were implicated with classical undertakings in carcinogenesis, especially in CRC (Figure 6D, Supplementary Table 5). Aberrant activation of WNT signaling pathway markedly increases the expression of β-catenin, thus promoting normal colon epithelial cells to infinitely proliferate and cancerate [25–27]. Moreover, TGF-beta/smad signaling pathway can advance invasion and metastasis of CRC by means of immune suppression, angiogenesis, augmenting interaction between tumor cells and extracellular matrix [28–30]. Overall, DE-lncRNAs and PCGs co-expression modules constructed by WGCNA were enriched in various pathways implicating important functions in regulating CRC progression.

**Table 4:** 10 LncRNAs with significant prognosis among five subtypes (including “normal group”).

**lncRNA**	**CNV frequency**	**PCC**	**Numberof DESubtype**	**p.value**	**GPL570.Probe**
LOC101927604	0.004425	0.121608	6	0.0457	1557702_at
LOC105377267	0.004425	0.065193	5	0.028027	NA
CASC15	0.006637	0.101133	6	0.031734	229280_s_at
LINC-PINT	0.002212	0.033925	5	0.013595	228702_at
CLDN10-AS1	0.037611	0.18445	6	0.017802	1570291_at
C14orf132	0.004425	0.044214	6	0.003379	231859_at
LMF1	0.00885	0.027102	6	0.031878	46142_at
LINC00675	0.019912	0.340102	6	0.039658	215658_at
CCDC144NL-AS1	0.011062	0.082301	5	0.009727	229669_at
LOC284454	0.011062	0.156642	5	0.006193	NA

### Identification of CNVs associated lncRNAs in CRC

**Figure 7:**
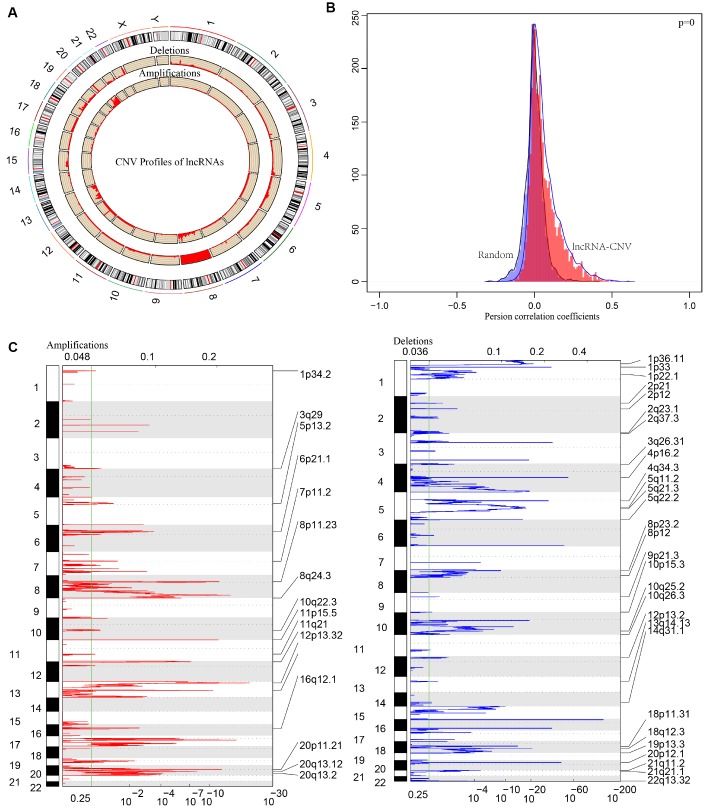
**The pattern of CNVs profiles in the whole genomes.** (**A**) The proportional frequencies of copy number deletions or amplifications of lncRNAs existing in the whole genome. CNV deletions of lncRNAs are mainly concentrated on 8 chromosome, and amplifications are focused on 8, 13, 20 chromosomes. The frequency of deletions are largely general than amplifications. (**B**) Distribution of correlation coefficient between copy number alternations of lncRNAs and the expression level of lncRNAs is shown. The correlation coefficient of CNV-lncRNA greater than 0 represents the regulation relationship is positive. (**C**) LncRNAs located in the focal peaks are CRC-related. False-discovery rates (q values) and scores from GISTIC 2.0 for alterations are plotted in x-axis, and the genome positions are shown as y-axis; dotted lines indicate the centromeres (distinguishing chromosome long arm from short arm). Amplifications (left, red) and deletions (right, blue) of lncRNAs are also shown. The green line represents 0.25 q value cut-off point that determines significance.

CNV is a frequent form of genomic structural changes, which is closely related to occurrence and deterioration of tumors. We profiled the spectrum of CNV-related lncRNAs on the whole genome in CRC patients The frequency of copy number deletions were largely more than amplifications, suggesting copy number deletions may predominantly implicate in CRC, and majority deletions were found on chromosome 8, in contrast, the amplifications were mainly concentrated on chromosome 20 (Figure 7A). The correlation between the expression profiles of lncRNAs and CNVs was demonstrating a positive relationship and the distribution was significantly higher than the random, p<1e-16 (Figure 7B). Moreover, the frequently changed regions in CRC genome were identified on the basis of GISTIC algorithm. A genome-wide view of the CNVs was shown in Figure 7C and the overall frequent change regions were complex. The focal amplification events (indicated by dashes), surpassing the significance threshold (green line), mainly distributes among chromosomes 8, 11, 12p13, 16q12, 20p11, and the focal deletions events were concentrated on 1p33, 3q26, 4p16, 5q11, 5q22, 20p12. Similarly to previous condition, frequent copy number deletions of lncRNAs were largely more than the copy number amplifications in the whole genome. This finding emphasized the impact of copy number deletions of lncRNAs in CRC, and it may be significantly related with prognosis of CRC patients.

**Figure 8:**
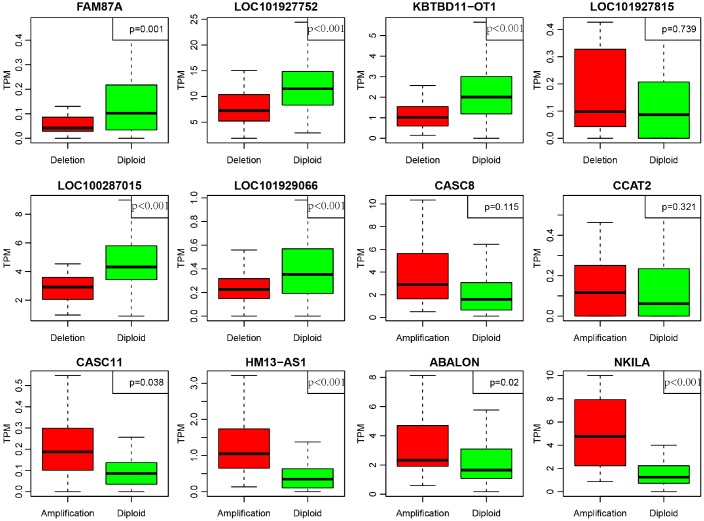
**Deregulation of lncRNAs induced by copy number deletions or amplifications (P<0.05).** Green (diploid) represents normal copy. Red (deletion or amplification) represents the variant copy.

In order to deeply explore the effect of CNV on transcription of lncRNAs, we screened out 17 lncRNAs with more than 7% of the frequency of CNV in 452 CRC samples (Supplementary Table 6). In the light of these 17 lncRNAs, we analyzed the expression difference of each lncRNA between the samples with copy deletion or copy amplification and normal group. A total of 12 lncRNAs were obtained according to the criterion in which the expression in each group was greater than 0. There are four lncRNAs: CASC11, HM13-AS1, ABALON, NKILA highly expressed in amplification group than diploid group (p<0.05), and FAM87A, LOC101927752, KBTBD11-OT1, LOC100287015, LOC101929066, these five lncRNAs were low-expressed in copy number deletion group than normal copy number group (p<0.05) (Figure 8). All these findings indicated that deletions or amplifications of lncRNAs may closely conduct the expression level of lncRNAs. However, these differentially expressed lncRNAs are deserved to explore for the potential clinical significance based on large-scale samples supporting.

### LncRNAs-based prognostic biomarkers in CRC patients

**Figure 9:**
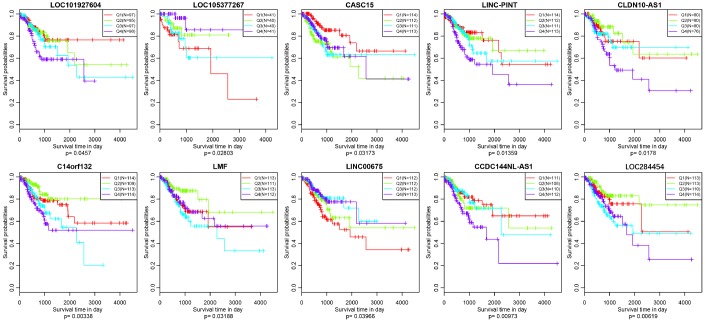
**Kaplan–Meier plot analysis shows disease-free survival (DFS) based on quartile method in TCGA (p<0.05).**

As we all know, lncRNAs are holding diving or inhibiting role in tumors and increasing numbers of lncRNAs have been conducted as prognosis associated biomarkers in CRC. In our research, we wondered whether the expression profiles of lncRNAs regulated by copy number deletions or amplifications can impact survival status of CRC patients. Based on the previous five molecular subtypes, we systematically analyzed the copy number alternations of these DE-lncRNAs among five subtypes, and then, 104 lncRNAs conforming to the following criterions were obtained (Supplementary Table 7): CNV change frequency among each sample is more than 0.1%; holding difference at least five subtypes; positively related with CNVs. We then wiped off the samples of whom the expression of each lncRNA was under zero, and subsequently correlated lncRNAs with disease-free survival. We finally acquired 10 lncRNAs with significant prognosis (p<0.05) based on the quartiles method (Figure 9, Supplementary Table 8). LOC101927604, CASC15, CLDN10-AS1, C14orf132, LMF1 and LINC00675 were differentially expressed among all six subtypes (including normal group), and the expression levels of LOC105377267, LINC-PINT, CCDC144NL-AS1 and LOC284454 were differential in five subtypes (Table 4).

**Figure 10:**
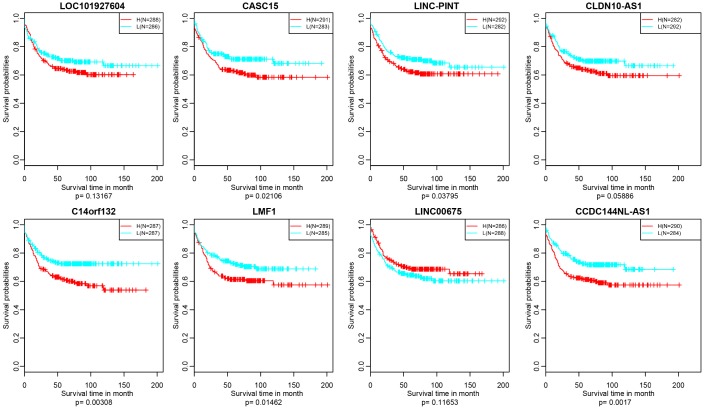
**Kaplan–Meier plot analysis shows relapse-free survival (RFS) validated in GEO datasets (p<0.05).**

In addition, we verified the prognostic value of identified 10 lncRNAs in GEO (Gene Expression Omnibus) dataset (GSE39582) based on GPL570 platform (https://www.ncbi.nlm.nih.gov/geo/). There were about 8 lncRNAs successfully annotated to probes existing in GEO platform and only 5 lncRNAs, CASC15, LINC-PINT, C14orf132, LMF1 and CCDC144NL-AS1 holding significant prognosis difference (p<0.05) (Figure 10).

## DISCUSSION

With next-generation sequencing and mass spectrometry technology sprouting quickly, biological complexity of tumors has been illuminated progressively. Previous knowledge have proved integrative clustering managements are capable to distinguish heterogeneity of tumors and to disclose practical prognostic signatures. The general integrative clustering methodologies include iCluster [19], intNMF (non-negative matrix factorization)[31], similarity network fusion (SNF)[32]. In this study, we firstly performed multi-dimensional data process according to iCluster which is based on a joint latent variable model in CRC. The most attractive signature of iCluster is that incorporates unobserved variables estimated from copy number data, mRNA expression data, methylation and others, simultaneously reducing the dimensionality of the datasets without changing sample size. A final cohort of 289 CRC patients were available for our algorithm matrix, and three omics data were covered by genomic and epigenomic, including mRNA sequencing, CNV and methylation downloading from TCGA, respectively. The integrative analysis unveiled five subtypes with distinctive molecular signatures and prognostic relevance. C3 subtype is exhibiting terrible prognosis and C2 subtype holds the most satisfactory outcome, which is extremely corresponding to distribution of TNM stage. A recent investigation has shown that copy number correlated genes (CNVcor) and methylation correlated genes (METcor) are co-regulated significantly and integration of CNVcor and METcor genes revealed three molecular subtypes of liver cancer [33]. Glioblastoma multiforme was subdivided into three molecular subtypes, namely subtype 1, subtype 2 and subtype 3 based on aggregation of DNA copy number, methylation and gene expression levels. Subtype 1 is characterized by hypermethylation involved in brain development and neuronal differentiation, and subtype 2 is presenting highly promoter methylation of homeobox and G-protein signaling genes, then, subtype 3 is featured with NF1 and PTEN alterations [34]. In our research, we also compared the mutation profiles of five molecular subtypes, and four mutation-related genes attracted our attention, TTN, APC, TP53 and KRAS, which are holding the highest mutation rate almost existing across five subtypes. Exome sequencing was performed to identify recurrent somatic mutations with prognostic significance and eventually APC, TP53 and KRAS were successfully diagnosed as mutation signatures in CRC [35].

The onset and progression of carcinogenesis is usually involved with thousands of genomic variants, including small-size mutations (SNPs) and large-scale genomic changes (CNVs), such as copy number deletions, duplications or amplifications. CNVs, hallmarks of cancer, often lead to aberrance in copy numbers, including amplification, gain, loss and deletion. CNVs are taking serious responsibility in regulating expression of PCGs and non-coding RNAs and the activations of multiple signaling pathways. It has been well-known that CNVs may conduct significant effects on various tumorigenesis, such as ovarian cancer [36], bladder cancer [37], hepatocellular carcinoma [38] and so on. Early stages (stages I and II) CRC were obviously exhibiting the most frequent deletions involved in chromosomes 6, 8p, 14q and 1p and the most frequent amplifications were mainly located in chromosome 19, 5, 2, 9p and 20p according to detection of CNVs in plasma [39]. The expression level and copy number of UQCRB protein (ubiquinol cytochrome c reductase binding protein), implicated in mitochondrial complex III stability, were unanimously upregulated in CRC, supporting CNVs induced deregulation effect [40]. Our study established novel regulatory factors in CRC, and lncRNAs (CASC11, HM13-AS1, ABALON, NKILA, FAM87A, LOC101927752, KBTBD11-OT1, LOC100287015, LOC101929066) were acting as new candidates for CRC diagnosis. CASC11 is an oncogenic lncRNA, which involved with influencing tumor cell stemness, cancer cell proliferation and epithelial-mesenchymal transition (EMT) in CRC, small cell lung cancer, bladder cancer, prostate cancer, hepatocellular carcinoma (HCC) [41–45]. NKILA, an NF-κB-interacting lncRNA, is determined for tumor-mediated T cell AICD (Activation-induced cell death) by inhibiting NF-κB activity, and NKILA overexpression in tumor-specific cytotoxic T lymphocytes (CTLs) is correlated with increased apoptosis and shorter survival in lung cancer and breast cancer [46]. Thus, we proposed a hypothesis that copy number of NKILA will be significantly amplified when CTLs are activated in CRC, following the increased expression of NKILA. We introduced a new mechanism underlying deregulated lncRNAs in tumorigenesis and the inspection of CNVs are convenient and precise.

Here, we described a picture for prognosis prediction of CRC with respect to CNVs relevant lncRNAs. In our research, we identified 10 lncRNAs associated with crucial clinical outcome in CRC, of which 5 lncRNAs (CASC15, LINC-PINT, C14orf132, LMF1 and CCDC144NL-AS1) were validated in GEO datasets. Cancer susceptibility candidate 15 (CASC15) is familiarly observed to participate in regulation of tumor proliferation, metastasis, and worsen survival probability of human cancer including CRC, cervical cancer, breast cancer and so on [47–49]. Jing et all demonstrated that CASC15 advances proliferation and metastasis on the basis of activating Wnt/βcatenin signaling pathway [50], and some other examinations displayed CASC15 may act as an oncogene, performing a pushing role in the progression of HCC, and upregulated expression of CASC15 is tied with imperfect prognosis [51]. Notwithstanding, copy number changes associated with CASC15 in tumors are far from being understood. p53-regulated human lncRNA (LINC-PINT) has been noticed functioning as a tumor suppressor by impeding proliferation of cancer cells in gastric cancer and glioblastoma [52, 53], and down-regulated LINC-PINT in pancreatic tumor may deliver unsatisfactory outcome [54]. C14orf132 (chromosome 14 open reading frame 132) gene is a novel long non-coding RNA (lincRNA) with unknown functions implicated in tumors, and the only investigation elucidated in C14orf132 is concerned with extremely low birth weight [55]. Lipase maturation factor 1 (LMF1) is a profound regulator of plasma lipid metabolism and majority studies mainly focused on mutations of LMF1 determining severe hypertriglyceridemia [56]. Up to now, there is still lacking of evidence about the latent position of LMF1 in tumorigenesis. Our inspection is the forerunner for exploring the prognostic value of LMF1 in CRC patients. CCDC144NL-AS1 is upregulated in ectopic endometrial (EC) tissues than eutopic endometrial (EU) tissues, and simultaneously exhibits elevated expression in advanced EC tissues (III+IV) [57]. Nevertheless, the distinct significance of CCDC144NL-AS1 in CRC is still inexperienced. Despite the fact that, these mentioned lncRNAs are prone to govern prognosis prediction among multiple tumors, prognostic signatures of lncRNAs guided by copy number changes remains elusive. The five prognostic lncRNAs are deserved to be furtherly verified on the foundation of large-scale clinical CRC samples.

In summary, systemic administration of RNA-seq, methylation and CNVs data presents original subtype classification methodology of CRC. Identification of deregulated lncRNAs induced by CNVs introduces an unprecedented regulatory pattern involved with lncRNAs, and prognosis prediction for CNV-related lncRNAs may take insight into precision diagnostics and therapeutics for CRC patients.

## METHODS

### Data preparation and processing

Involved original data were downloaded from official TCGA data portal (https://tcga-data.nci.nih.gov), including RNA sequencing data, 450k methylation data, CNV data, DNA mutation data and clinical information. Subsequently, series of managements were performed, for instance, RNA sequence data: primary data were downloaded in “counts” form, and the processed FPKM (Fragments Per Kilobase of exon model per Million mapped fragments) data were transformed to TPM (TranscriptsPerKilobase of exonmodel per Million mapped reads). The gene expression profiles of 458 CRC patients and 41 normal cases were acquired. Then, we defined lincRNA, sense-intronic, sense-overlapping, antisense, processed-transcript, 3primer-overlapping as lncRNAs based on the genecode file. Meanwhile, gene type belonging to protein-coding was assigned to PCGs; Next, administrations of 337 CRC patients holding 450k methylation data were involved with removing of NA probes, cross-reactive CpG sites [18] and unstable CpG sites existing in the sex chromosomes and single nucleotide sites; Then, CNV data and single nucleotide mutation data were pictured, with the removed germline difference and processed by “mutect software”, respectively. Eventually, we also obtained corresponding clinical information of 458 CRC patients. The patients with following time less than 30 days were removed. Underlying these prepared data, we employed R package-iCluster to integrate PCGs, methylation and CNVs for subtypes clustering. Number of clusters was set to 5 and 50 iterations were set to identify stable samples clusters with default parameters [19]. The R code can be downloaded at http://www.mskcc.org/mskcc/html/85130.cfm.

### Differentially expressed lncRNAs and mRNAs across five subtypes

The R package-DEseq2 was claimed for determining these differentially expressed lncRNAs and mRNAs between five subtypes and normal tissues specimens, with the criteria of fold change>2, FDR<0.05. Genes with count<1 were rejected among the whole genome profiles. The basic data structure consisted of two tables, including countData and colData. Gene set enrichment analysis (GSEA) was furtherly applied to distinguish the distribution of DE-lncRNAs and the difference multiple was used for ranking.

### Weighted gene co-expression network analysis (WGCNA) to identify phenotypes-related modules

WGCNA is often regarded as efficient methodology applying in picking up co-expressed gene modules and identifying potential therapeutic targets associated with clinical phenotypes [20]. In present research, based on the differential expression profiles of lncRNAs and PCGs, co-expression network was constructed using WGCNA. In order to make network conform to non-scale signature, the soft threshold was defined as 3. The non-scale signature means that log(k) is negatively correlated with log(P(k)), and the correlation coefficient is greater than 0.8. The possible modules were identified with the average-linkage clustering and dynamic tree cut on the ground of height=0.25, deepSplit = 2, minModuleSize = 30. Considering the clinical information downloading from TCGA, the correlation between each module and corresponding clinical information was calculated. Immediately, annotations of potential pathways were targeted in order to screen out biologically interested modules based on the userListEnrichment function included in package WGCNA [21].

### Copy number expression profiles of whole genomic lncRNAs

GISTIC (Genomic Identification of Significant Targets in Cancer) algorithm is responsible for identifying variant regions that are more prone to drive cancer pathogenesis [22]. It can visualize regions in the genome manifesting amplifications or deletions across thousands of samples. G-score is allocated to each alternational region to evaluate the amplitude of aberration and the frequency of occurrence [23]. Here, GISTIC2.0 software was applied to define the prepared CNVs profiles of all genes underlying 452 CRC samples, and lncRNAs-related CNVs expression profiles were extracted. We defined copy number>1 or <-1 as copy number amplifications or deletions respectively. False Discovery Rate (FDR) q-values were assigned to each alternational region. “Peak regions”, also known as significantly aberrant regions, point to the greatest frequency and amplitude of aberrations. The “peak regions” aim at determining whether the signal is primarily due to broad events (longer than half a chromosome arm), focal events, or significant levels of both [23, 24].

### CNV-related lncRNAs acting as prognostic biomarkers in CRC patients

CNVs profiles of DE-lncRNAs among five subtypes were described. Associated lncRNAs were prepared with the criterions as following: 1) the identified lncRNAs must be positively correlated with CNVs (correlation coefficient>0); 2) the identified lncRNAs should have significant difference across at least 5 subtypes (number of DEsubtype≧5); 3) the identified lncRNAs hold copy number change ratio more than 0.1% in all involved cases (CNV frequency>0.001); 4) prognostic-related lncRNAs were marked with p<0.05 (samples were divided into four groups based on quartile method or two groups based on median level). The selected patients must manifest expression value of previous marked lncRNAs greater than 0.

### Statistical analysis

All statistical strategies in our research were based on R 3.4.3 (https://cran.r-project.org/bin/windows/base/old/3.4.3/). All workings are dependent on default parameters except to special notes.

## Supplementary Material

Supplementary Figures

Supplementary Tables

Supplementary Table 1

Supplementary Table 2

Supplementary Table 4

Supplementary Table 6

Supplementary Table 7

Supplementary Table 8
