# Supplementary figures and images for: Copy number variations primed lncRNAs deregulation contribute to poor prognosis in colorectal cancer
Source: Aging (Albany NY). 2019 Aug 22;11(16):6089–108. doi: 10.18632/aging.102168 (PMC6738420; doi:10.18632/aging.102168)

SUPPLEMENTARY FIGURES

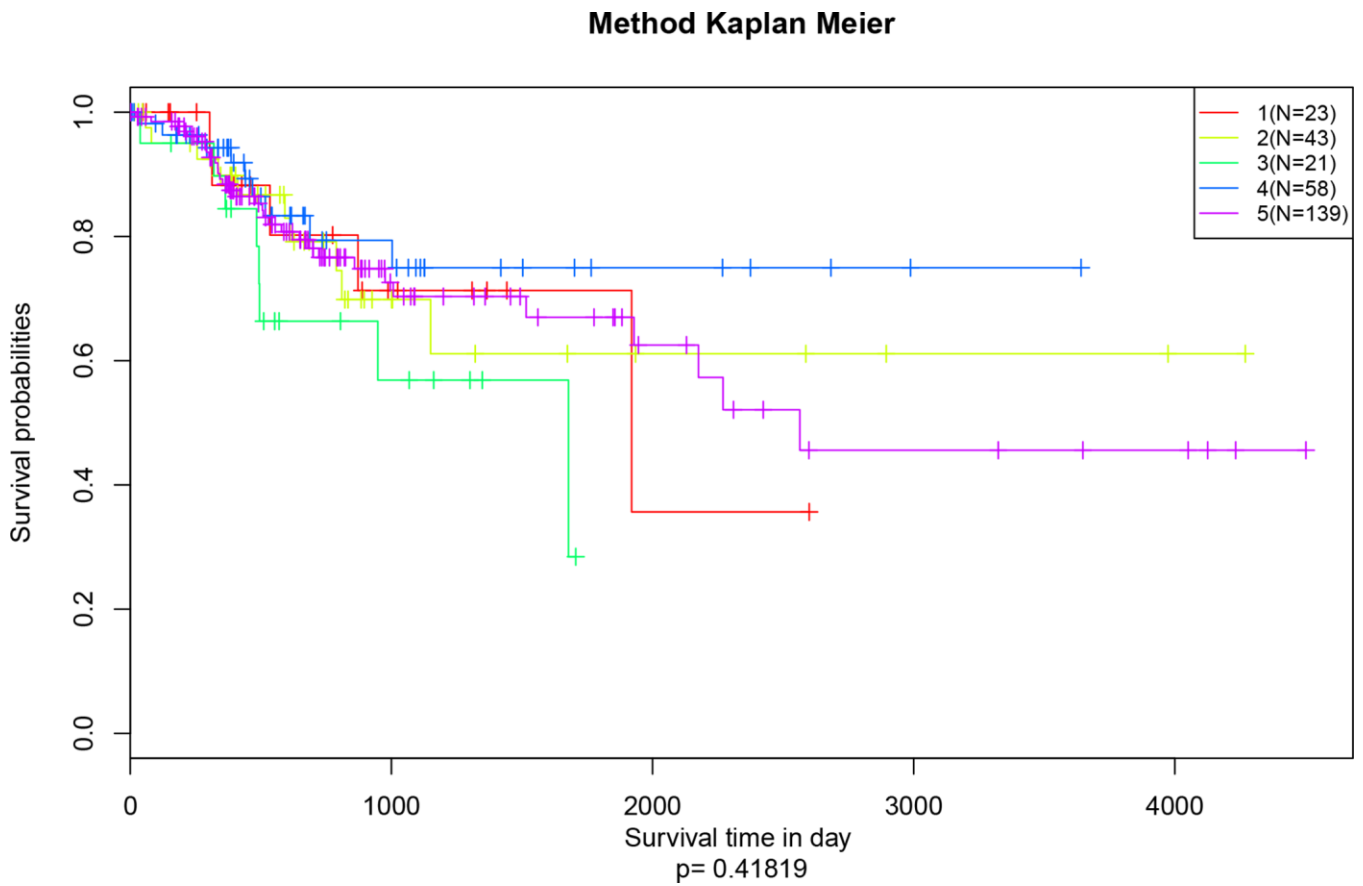

Supplementary Figure 1.

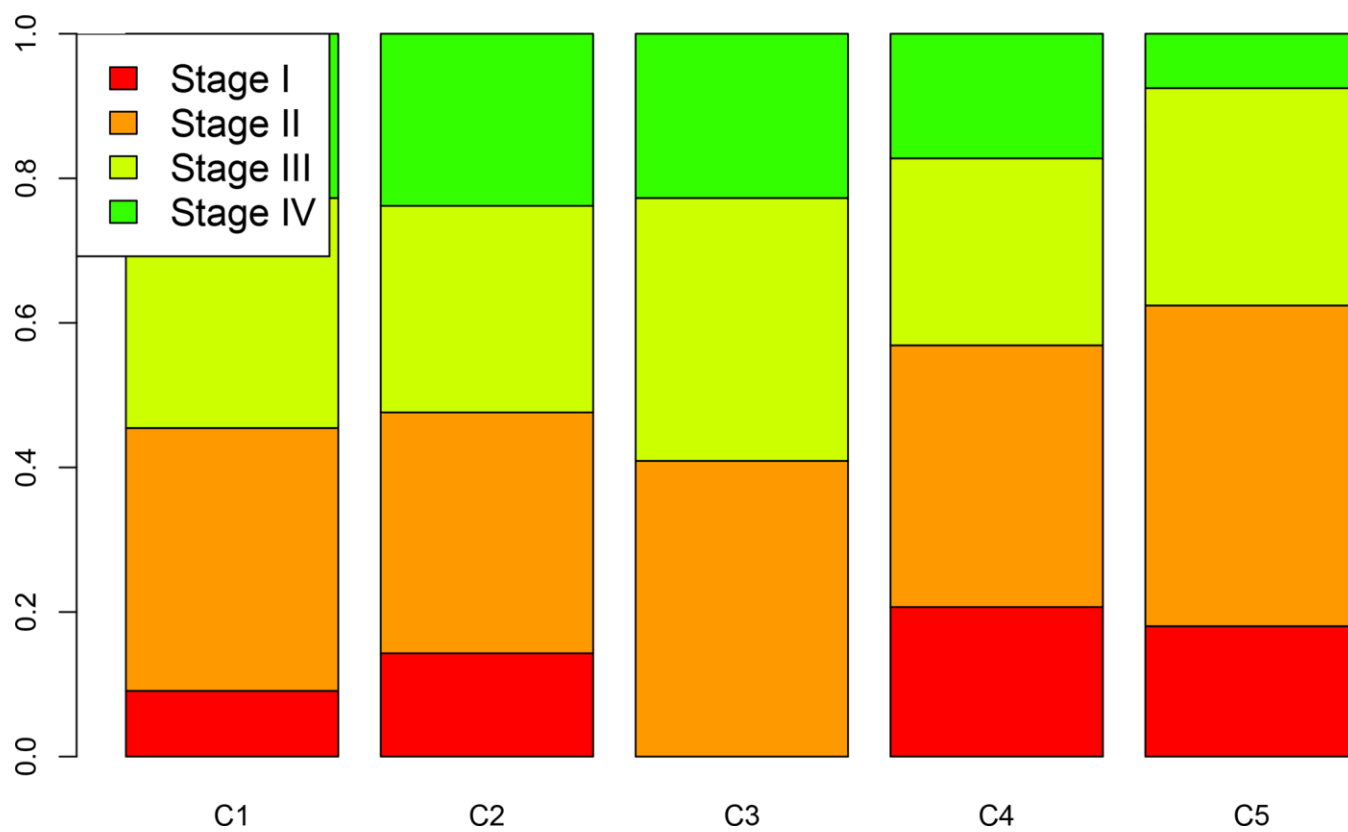

Supplementary Figure 2.

Supplement: Supplementary Figures [file aging-11-102168-s008.pdf]
