# Supplementary material for: Copy number variations primed lncRNAs deregulation contribute to poor prognosis in colorectal cancer
Source: Aging (Albany NY). 2019 Aug 22;11(16):6089–108. doi: 10.18632/aging.102168 (PMC6738420; doi:10.18632/aging.102168)
Supplement: Supplementary Tables [file aging-11-102168-s007.pdf]

SUPPLEMENTARY TABLES

Please browse Full Text version to see the data of Supplementary Tables 1 and 2.

Supplementary Table 1.

Supplementary Table 2.

Supplementary Table 3.

ID	Description	GeneRatio	BgRatio	pvalue	p.adjust	qvalue	geneID	Count
hsa05206	MicroRNAs in cancer	11841	299/7466	0.001473	0.117837	0.117837	VEGFA/MIR25/ MIR200A/MIR135B/ MIR200B/MIR199A1	6
hsa04931	Insulin resistance	11749	107/7466	0.010498	0.419906	0.419906	MLXIPL/OGT/CPT1B	3

Please browse Full Text version to see the data of Supplementary Table 4.

Supplementary Table 4.

Supplementary Table 5.

ID	Description	GeneRatio	BgRatio	pvalue	p.adjust	qvalue	geneID	Count
hsa04926	Relaxin signaling pathway	26420	130/7466	0.008169	0.38588	0.365784	RLN1/ADCY3/ PLCB4/RLN2/ GNG4	5
hsa03022	Basal transcription factors	26359	45/7466	0.009133	0.38588	0.365784	TAF4/GTF2F2/ GTF2IRD1	3
hsa00510	N-Glycan biosynthesis	26359	49/7466	0.011538	0.38588	0.365784	RPN2/ALG5/ MGAT5	3
hsa05110	Vibrio cholerae infection	26359	50/7466	0.012191	0.38588	0.365784	PLCG1/ADCY3/ ATP6V1C2	3
hsa04713	Circadian entrainment	26390	96/7466	0.013566	0.38588	0.365784	ADCY3/PLCB4/ CACNA1D/ GNG4	4
hsa04310	Wnt signaling pathway	26420	149/7466	0.014186	0.38588	0.365784	AXIN2/CSNK2A2/ TCF7/PLCB4/ NKD1	5
hsa04916	Melanogenesis	26390	101/7466	0.016087	0.38588	0.365784	ADCY3/TCF7/ PLCB4/ASIP	4
hsa04934	Cushing syndrome	26420	154/7466	0.016163	0.38588	0.365784	ADCY3/AXIN2/ TCF7/PLCB4/ CACNA1D	5

hsa04725	Cholinergic synapse	26390	112/7466	0.02262	0.390425	0.370091	ADCY3/PLCB4/ CACNA1D/GNG4	4
hsa04927	Cortisol synthesis and secretion	26359	64/7466	0.023557	0.390425	0.370091	ADCY3/PLCB4/ CACNA1D	3
hsa04724	Glutamatergic synapse	26390	114/7466	0.023959	0.390425	0.370091	ADCY3/PLCB4/ CACNA1D/GNG4	4
hsa04137	Mitophagy - animal	26359	65/7466	0.024529	0.390425	0.370091	BCL2L1/ATG9B/ CSNK2A2	3
hsa00562	Inositol phosphate metabolism	26359	74/7466	0.034249	0.487047	0.461681	PLCG1/PLCB4/ INPP5D	3
hsa05016	Huntington disease	26420	193/7466	0.038044	0.487047	0.461681	TAF4/SDHA/ PLCB4/ATP5A1/ DNAH14	5
hsa04915	Estrogen signaling pathway	26390	137/7466	0.042819	0.487047	0.461681	ADCY3/PLCB4/ KRT23/KRT39	4
hsa04146	Peroxisome	26359	83/7466	0.045682	0.487047	0.461681	ECH1/PIPOX/ MPV17L	3
hsa03030	DNA replication	26330	36/7466	0.046803	0.487047	0.461681	RNASEH2B/RFC3	2
hsa04350	TGF-beta signaling pathway	26359	85/7466	0.04845	0.487047	0.461681	TFDP1/TGIF2/ NODAL	3
hsa04911	Insulin secretion	26359	85/7466	0.04845	0.487047	0.461681	ADCY3/PLCB4/ CACNA1D	3

Please browse Full Text version to see the data of Supplementary Tables 6–8.

Supplementary Table 6.

Supplementary Table 7.

Supplementary Table 8.
